# Additive Value of Dynamic FDOPA PET/CT for Glioma Grading

**DOI:** 10.3389/fmed.2021.705996

**Published:** 2021-07-09

**Authors:** Antoine Girard, Pierre-Jean Le Reste, Alice Metais, Nibras Chaboub, Anne Devillers, Hervé Saint-Jalmes, Florence Le Jeune, Xavier Palard-Novello

**Affiliations:** ^1^Univ Rennes, CLCC Eugène Marquis, Noyau Gris Centraux EA 4712, Rennes, France; ^2^CHU Rennes, Rennes, France; ^3^Univ Rennes, CLCC Eugène Marquis, INSERM, LTSI – UMR 1099, Rennes, France

**Keywords:** FDOPA, positron-emission tomography, gliomas grading, dynamic, quantification

## Abstract

**Purpose:** The aim of this study was to assess the value of the FDOPA PET kinetic parameters extracted using full kinetic analysis for tumor grading with neuronavigation-guided biopsies as reference in patients with newly-diagnosed gliomas.

**Methods:** Fourteen patients with untreated gliomas were investigated. Twenty minutes of dynamic positron-emission tomography (PET) imaging and a 20-min static image 10 min after injection were reconstructed from a 40-min list-mode acquisition immediately after FDOPA injection. Tumors volume-of-interest (VOI) were generated based on the MRI-guided brain biopsies. Static parameters (TBRmax and TBRmean) and kinetic parameters [K1 and k2 using full kinetic analysis with the reversible single-tissue compartment model with blood volume parameter and the time-to-peak (TTP)] were extracted. Performances of each parameter for differentiating low-grade gliomas (LGG) from high-grade gliomas (HGG) were evaluated by receiver-operating characteristic analyses (area under the curve; AUC).

**Results:** Thirty-two tumoral VOI were analyzed. K1, k2, and TTP were significantly higher for HGG than for LGG (median K1-value = 0.124 vs. 0.074 ml/ccm/min, *p* = 0.025, median k2-value = 0.093 vs. 0.063 min^−1^, *p* = 0.025, and median TTP-value = 10.0 vs. 15.0 min, *p* = 0.025). No significant difference was observed for the static parameters. The AUC for the kinetic parameters was higher than the AUC for the static parameters (respectively, AUC_K1_ = 0.787, AUC_k2_ = 0.785, AUC_TTP_ = 0.775, AUC_TBRmax_ = 0.551, AUC_TBRmean_ = 0.575), significantly compared to TBRmax (respectively, *p* = 0.001 for K1, *p* = 0.031 for k2, and *p* = 0.029 for TTP).

**Conclusion:** The present study suggests an additive value of FDOPA PET/CT kinetic parameters for newly-diagnosed gliomas grading.

## Introduction

Gliomas are the most commonly occurring primary malignant brain tumor in adults (1). Patient outcome and treatment strategy are still mainly defined by tumor grade according to the World Health Organization (WHO) classification (2). Furthermore, the main goal of the gliomas surgical resection is to remove as much of the tumor as safely achievable (2, 3). So, in order to determine the extent of tumor resection, presurgical identification of high-grade subregions is needed. Currently, magnetic resonance imaging (MRI) with contrast-enhanced (CE), diffusion, and perfusion sequences is the imaging method of reference for gliomas grading (2). However, around one quarter of low-grade gliomas (LGG) present contrast-enhancement (4) and one-third of non-enhancing tumors are high-grade gliomas (HGG) (5). In order to improve gliomas grading, amino-acid (AA) positron-emission tomography (PET) tracers including O-(2-[18F]-fluoroethyl)-L-tyrosine (FET), [11C]-methionine (MET), and 3,4-dihydroxy-6-[18F]fluoro-L-phenylalanine (FDOPA) have been increasingly investigated (6). To date, FET is the most studied AA PET tracer. However, FDOPA is the only AA PET tracer widely available in several countries (7). FDOPA is transported into the cells mainly by L-type amino acid transporter 1 (LAT 1), which is overexpressed in gliomas (6). Regarding performances of FDOPA PET/CT for gliomas grading, previous studies have provided conflicting results (8–18). However, almost all of these studies used static parameters for FDOPA uptake quantification. Advanced pharmacokinetic analysis of time-activity curves (TAC) from dynamic PET scans using compartment models enables the extraction of direct physiological parameters and could add further information concerning tumor aggressiveness for several cancers (19, 20). Positron-emission tomography kinetic analysis has recently increased with newly developed PET systems that offer higher count rate capabilities than previous scanners. For gliomas assessment, studies shown that FET PET kinetic analysis can provide useful information about the tumor characteristics (21, 22), so dynamic analysis has been recommended in recent guidelines (6).

The aim of the present study was to assess the value of the FDOPA PET kinetic parameters parameters extracted using full kinetic analysis for tumor grading with neuronavigation-guided biopsies as reference in patients with newly-diagnosed gliomas.

## Materials and Methods

### Participants

From June 2018 to September 2019, 19 patients with suspected supratentorial diffuse gliomas were prospectively included in the “GLIROPA” clinical trial (NCT03525080). All patients were newly diagnosed for gliomas and selected for resective surgery. Included patients had to be at least 18 years-old and covered by national health insurance; and neither be pregnant, nor in an emergency situation, nor be treated by carbidopa, catechol-O-methyl transferase inhibitor, haloperidol, or reserpine medication. All patients provided their written informed consent. This study has been performed in accordance with the Declaration of Helsinki and approved by an independent national research ethics committee (CPPIDF1-2018-ND27-cat.2).

### PET/CT Imaging Protocol

The patients were required to fast at least 4 h before undergoing the imaging protocol. Unenhanced CT (2 mm reconstructed section thickness using iterative method, 512 × 512 matrix; pitch index, 0.55) was performed with automated tube current modulation (CARE Dose4D) and automated tube voltage selection (CARE kV) followed by a PET acquisition using list-mode acquisition with a single field of view centered on the brain (Siemens Healthcare Biograph mCT Flow, Erlangen, Germany). Hundred and forty-nine MBq (range 122–192) of FDOPA were slowly administered intravenously, without carbidopa premedication. Positron-emission tomography images were reconstructed with attenuation correction, without point-spread function correction, using a fully 3D ordered-subset expectation maximization algorithm (8 iterations and 21 subsets) with a 400 × 400, matrix and 4 mm kernel convolution filter. Voxel size (XYZ) was 1 × 1 × 2 mm^3^. A 20-min static image 10 min after injection in accordance with current recommendations (23) and an optimal dynamic time sampling of 8 × 15 s-−2 × 30 s-−2 × 60 s-−3 × 300 s from the bolus arrival time (24) were reconstructed from a 40-min list-mode acquisition immediately after FDOPA injection.

### Surgical Biopsies

Surgeries were performed under general anesthesia, with the aid of neuronavigation from StealthStation S7 (Medtronic, Dublin, Ireland). Up to three neuronavigation-guided biopsies of 1 cm^3^ were performed per patient, depending on the tumor size. Biopsy targets were located on 3D-T2 fluid-attenuated inversion recovery (FLAIR) weighted MR imaging, prospectively and previously defined with all of MRI sequences and FDOPA PET/CT imaging. Each resection specimen and biopsy sample was collected, prepared, and analyzed blind to imaging. Samples were prepared using standard histopathological techniques. Diagnosis and grading were performed on formalin-fixed paraffin-embedded tissue sections stained by hematoxylin and eosin, using complementary techniques to detect isocitrate dehydrogenase (IDH) mutation and 1p19q codeletion, in accordance with the World Health Organization (WHO) 2016 Classification of Tumors of the Central Nervous System (25). Each biopsy sample was graded independently. Grade biopsy results were grouped into LGG and HGG, including grade II for LGG, and grade III-IV for HGG, respectively. For each patient, the IDH genotype of the whole specimen was applied for each individual biopsy sample.

### Image Analysis

**Figure 1:**
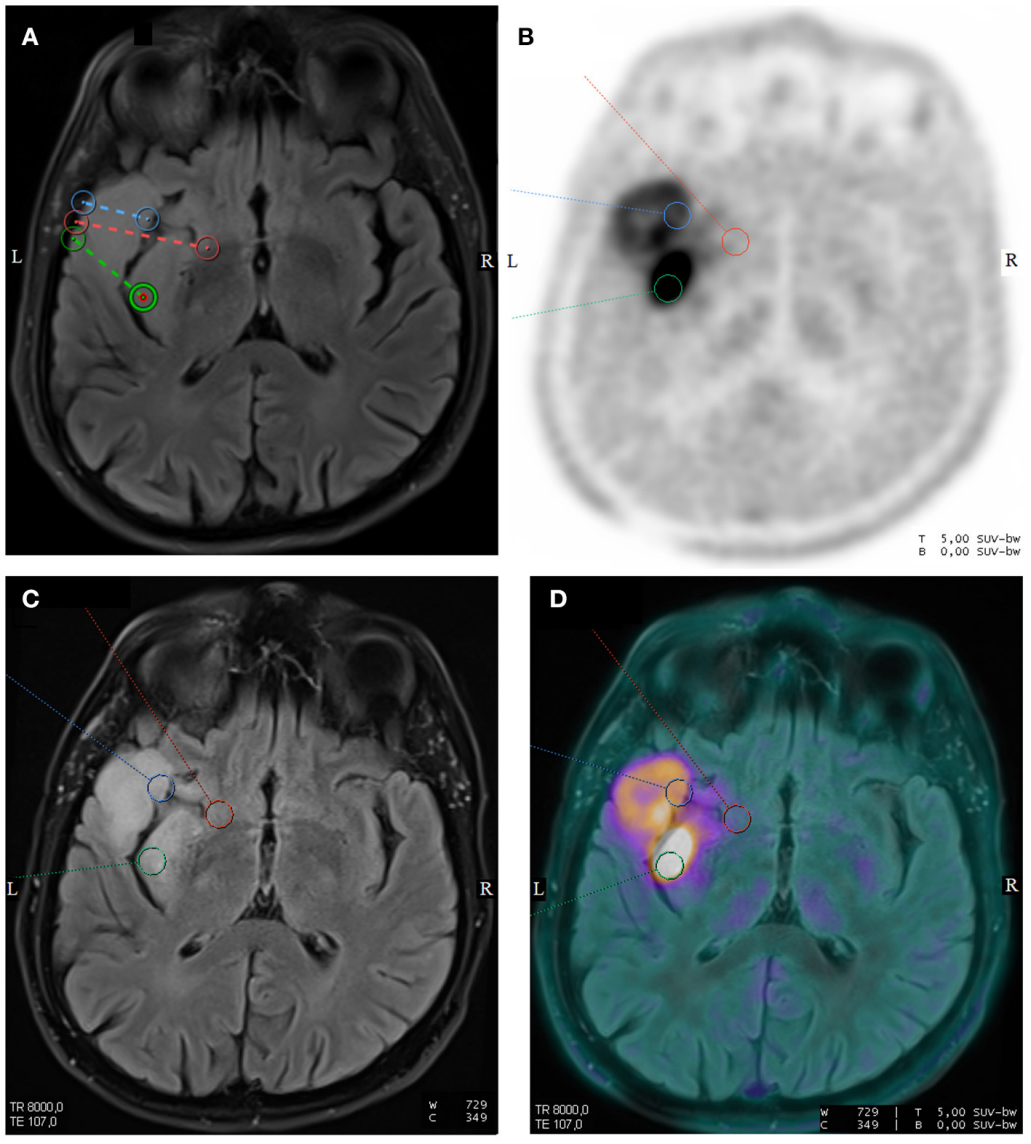
Axial T2-weighted fluid-attenuated inversion recovery (FLAIR) sequence from surgical navigation software with the locations of three neuronavigation-guided biopsies in a 59-year-old man with a left temporal diffuse IDH1-wild-type glioblastoma **(A)**. Corresponding axial images of FDOPA PET **(B)**, T2-weighted FLAIR sequence **(C)**, and fused FDOPA PET/T2-weighted FLAIR sequence (D) are displayed. The pathology grading of the neuronavigation-guided biopsies revealed WHO grade II for the red VOI and WHO grade III for the green and blue VOIs.

Volume-of-interest (VOI) of 1 cm^3^ were drawn on each biopsy site using the Syngo.via software (Siemens Healthcare), after registration with the FLAIR weighted images used for the neuronavigation-guided brain biopsies (Figure 1). For each voxel, the standardized uptake value (SUV) was calculated using the following formula: SUV = tissue radioactivity concentration/[injected activity/patient weight]. SUVmax and SUVmean were, respectively, the maximum and the mean of the SUVs of each VOI. A reference area was drawn on one slice including the whole normal contralateral hemisphere at the centrum semiovale level, for the computing of Tumor-to-normal brain (TBR) ratios. Tumor-to-normal brain ratio were computed as SUVmax or SUVmean of the VOI of each biopsy site by the SUVmean of the normal brain (TBRmax and TBRmean). Volume-of-interests of each biopsy site were projected onto each frame of the dynamic reconstruction. Time-to-peak (TTP), corresponding to the delay between the beginning of the acquisition and the timepoint of the maximal mean activity concentration for each VOI. On the early PET image with the maximum blood pool activity, a VOI was manually drawn into the middle cerebral artery to estimate an imaging-derived input function (IDIF). For each patient, FDOPA plasma input function was obtained after corrections for metabolites and hematocrit. Imaging-derived input function was fitted to the measured fractions of metabolites taken from the publication of Huang et al. (26). To extract kinetic parameters (PMOD software version 3.8; PMOD Technologies; Zürich, Switzerland), the reversible single-tissue compartment model with blood volume parameter (1T2k+VB, with K1 = rate constant from blood to tissue, k2 = rate constant from the tissue compartment to the arterial blood) was selected on the basis of the Akaike information criterion for small sample sizes (27).

### Statistical Analysis

To compare quantitative kinetic and static FDOPA PET parameters between LGG and HGG, to compare kinetic parameters between CE and non-contrast lesions, and to compare FDOPA PET parameters with gender, a Mann–Whitney U-test was performed. The diagnostic performances of these parameters to discriminate LGG from HGG were assessed by receiver-operator characteristic (ROC) curve analyses using histological grading as reference. The optimal thresholds were defined based on maximization of Youden's index. The area under the ROC curve (AUC) was also determined for each quantitative parameter. Accuracy for grading of the kinetic parameters and CE MRI were compared pairwise with the McNemar *t*-test. A Spearman rank correlation test was used for correlation analysis between FDOPA PET parameters and the age of the participants. A *p*-value <0.05 with false discovery rate adjustement for multiple comparisons was considered as significant. Statistical analysis was performed using MedCalc® version 12.5.0.0 (Medcalc Software bvba).

## Results

### Patients

Two patients were excluded because surgeries were performed in other hospitals. Another patient was also excluded because no surgical resection was performed. The dynamic acquisition was unsuccessful for two patients. Thirty-two biopsy sites from 14 patients were finally analyzed. Patient and tumor characteristics are set out in Table 1. There were eight men and six women, with a median age of 40 years (range 23–66). The distribution of the 14 cases was as follows: six astrocytomas-IDH-mutant; two oligodendrogliomas/1p19q-codeleted-IDH-mutant; and six IDH-wild-type glioblastomas. The MRI was performed within a median time of 3 days (range 1–23) after FDOPA PET/CT. Surgery was performed within a median time of 14 days (range 6–110) after FDOPA PET/CT.

**Table 1:** Summary of patient characteristics and imaging findings.

**Characteristic**	**Value**
Age (years), median (range)	40 (23–66)
Gender (*N* = 14)	
Male	9 (64%)
Female	5 (36%)
Tumor types (*N* = 14)	
Astrocytomas-IDH mutant	6 (43%)
Oligodendrogliomas/1p19q-codeleted-IDH mutant	2 (14%)
IDH-wild-type glioblastomas	6 (43%)
WHO grading of each biopsy sites (*N* = 32)	
II	9 (28%)
III	18 (56%)
IV	5 (16%)
K1 (ml/ccm/min), median (range)	0.103 (0.055–0.578)
k2 (min^−1^), median (range)	0.082 (0.027–0.180)
TTP (min), median (range)	15.0 (1.25–20.0)
TBR max, median (range)	2.3 (1.4–6.2)
TBRmean, median (range)	1.6 (0.8–5.0)

Among the 32 biopsy samples analyzed, nine (28%) were grade II, eighteen (56%) were grade III, and five (16%) were grade IV. The CE-MR sequence revealed that eight biopsy sites in five participants were CE and 24 biopsy sites in the other nine participants were not CE. Using a tumor-to-brain ratio higher than 1.7, in accordance with previously published data (17), 11 biopsy sites in seven participants were considered as increased FDOPA uptake and 21 biopsy sites in the other seven participants were not considered as FDOPA increased uptake.

### Comparison of Quantitative FDOPA PET/CT Parameters and Gliomas Grading

**Figure 2:**
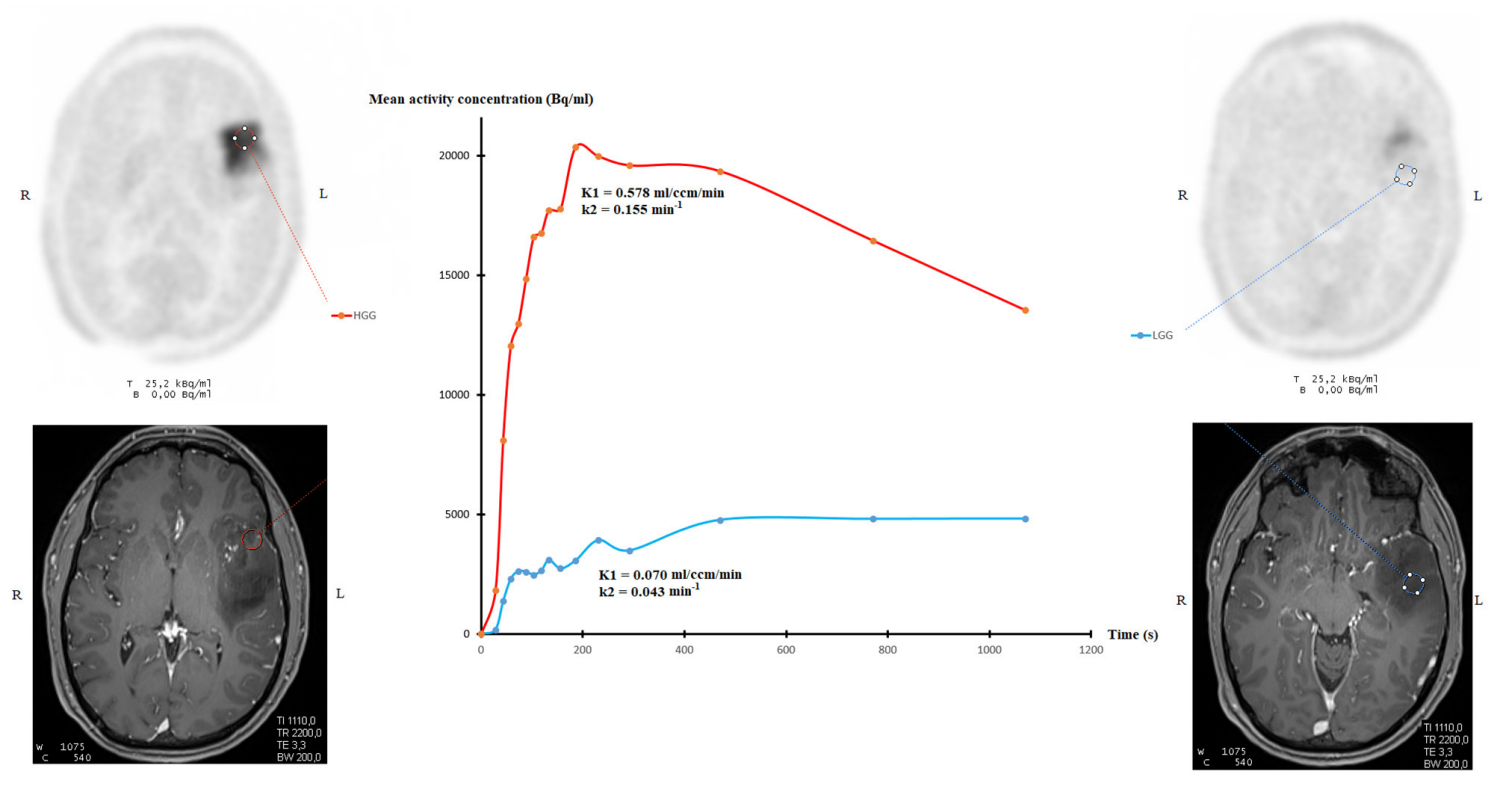
Examples of time-activity curves extracted from a HGG VOI (red line) and extracted from a LGG VOI (blue line) in a 27-year-old man with a left temporal WHO grade III astrocytoma IDH1-mutant, with corresponding CE-MRI image.

All quantitative FDOPA PET parameters are given in Table 1. K1 and k2 were significantly higher for HGG than for LGG and TTP was significantly lower for HGG than for LGG (Table 2). Examples of TAC extracted from a HGG VOI and TAC extracted from a LGG VOI in a 27-year-old man with a left temporal WHO grade III astrocytoma IDH1-mutant are shown in Figure 2. The AUC for the kinetic parameters was higher than the AUC for the static parameters, significantly compared to TBRmax (AUC_K1_ = 0.787 vs. AUC_TBRmax_ = 0.551, *p* = 0.001; AUC_k2_ = 0.785 vs. AUC_TBRmax_, *p* = 0.031 and AUC_TTP_ vs. AUC_TBRmax_, *p* = 0.029) (Table 3). K1, k2, and TBRmax were significantly higher for IDH-wildtype than for IDH-mutant and TTP was significantly lower for IDH-wildtype than for IDH-mutant (Table 4). Diagnostic accuracy of k2 was significantly higher than these with CE (accuracy for k2 = 0.63 vs. 0.47 for CE, *p* = 0.042). There was a trend toward greater K1 for CE biopsy sites than for non-CE (median K1-value was, respectively, 0.266 vs. 0.098 ml/ccm/min, *p* = 0.06). TBRmax, TBRmean, K1, and k2 were positively correlated with age (respectively, *r* = 0.54, *p* = 0.003; *r* = 0.41, *p* = 0.024; *r* = 0.38, *p* = 0.033; *r* = 0.54, *p* = 0.003) and TTP was negatively correlated with age (respectively *r* = −0.53, *p* = 0.003). There were no differences in static and kinetic parameters between female and male participants.

**Table 2:** Comparison of imaging parameters and WHO grade of each biopsy site.

**Parameters**	**WHO Grade**		***p-value***
	**LGG (*n* = 9)**	**HGG (*n* = 23)**	
median K1 (ml/ccm/min), range	0.074, 0.055–0.131	0.124, 0.056–0.578	0.025^*^
median k2 (min^−1^), range	0.063, 0.027–0.085	0.093, 0.043–0.180	0.025^*^
median TTP (min), range	15.0, 10.0–20.0	10.0, 1.25–20.0	0.025^*^
median TBRmax, range	2.2, 1.7–3.7	2.4, 1.4–6.2	***0.681***
median TBRmean, range	1.7, 1.2–2.7	1.6, 0.8–5.0	***0.670***

* *p-value < 0.05 with false discovery rate adjustment = statistically significant*.

**Table 3:** Diagnostic performances of FDOPA parameters for discrimination of LGG and HGG.

**Parameters**	**AUC** **(CI 95%)**	***p*-value**	**Threshold**	**Youden's index**	**Sensitivity** **(CI 95%)**	**Specificity** **(CI 95%)**	**Accuracy** **(CI 95%)**
K1^**^ (ml/ccm/min)	0.787 (0.607–0.911)	0.028^*^	0.131	0.48	0.48 (0.27–0.69)	1 (0.66–1)	0.63 (0.44–0.79)
k2^**^ (min^−1^)	0.785 (0.604–0.910)	0.028^*^	0.085	0.61	0.61 (0.39–0.80)	1 (0.66–1)	0.72 (0.53–0.86)
TTP^**^ (min)	0.775 (0.594–0.903)	0.028^*^	13.3	0.50	0.61 (0.39–0.80)	0.89 (0.52–1)	0.69 (0.50–0.84)
TBRmax	0.551 (0.366–0.726)	0.104	2.7	0.32	0.43 (0.20–0.59)	0.89 (0.52–1)	0.56 (0.38–0.74)
TBRmean	0.575 (0.388–0.747)	0.104	2.0	0.19	0.30 (0.13–0.53)	0.89 (0.52–1)	0.47 (0.29–0.65)

* *p-value < 0.05 with false discovery rate adjustment = statistically significant for AUC compared to 0.500*.

** *p-value < 0.05 with false discovery rate adjustment = statistically significant for pairwise comparison of ROC curves with TBRmax*.

**Table 4:** Comparison of imaging parameters and IDH mutation status of each biopsy site.

**Parameters**	**IDH mutation status**		***p-value***
	**IDH-mutant (*n* = 16)**	**IDH-wildtype (*n* = 16)**	
median K1 (ml/ccm/min), range	0.088, 0.055–0.578	0.154, 0.067–0.452	0.049^*^
median k2 (min^−1^), range	0.064, 0.027–0.155	0.091, 0.046–0.180	0.038^*^
median TTP (min), range	15.0, 3.0–20.0	7.5, 1.25–20.0	0.003^*^
median TBRmax, range	2.1, 1.4–6.2	3.0, 1.5–5.1	0.049^*^
median TBRmean, range	1.5, 0.8–5.0	1.7, 1.0–4.0	***0.160***

* *p-value < 0.05 with false discovery rate adjustment = statistically significant*.

## Discussion

Parameters extracted from FDOPA PET full kinetic analysis are well-associated with tumoral aggressiveness. To the best of our knowledge, this is the first study comparing FDOPA uptake kinetic parameters and pathology grading with biopsy validation. The characterization of gliomas with full kinetic analysis using compartmental modeling has been reported in two previous studies but serial biopsies were not performed. Schiepers et al. showed that K1 was higher in HGG than LGG in a pilot study including nine patients with newly diagnosed gliomas (18). Nioche et al. also displayed that the use of K1 enables differentiation between LGG and HGG in a study including 20 patients with newly diagnosed gliomas (15). The findings of our prospective biopsy-controlled study are consistent with these two previous researches. More recently, using simplified kinetic analysis with determination of SUV at different time points, Ginet et al. showed that IDH-wildtype gliomas, which are associated with a poorer outcome (28), have a shorter TTP than IDH-mutant gliomas (13). In that study, authors also reported that IDH-wildtype gliomas have a higher negative slope (linear regression applied on the 10th to 30th minute interval of SUVmean-based curve) than IDH-mutant gliomas. Our results support that higher FDOPA uptake rate constant and FDOPA clearance rate constant might serve as non-invasive markers of aggressiveness. Moreover, we found that accuracy for glioma grading of k2 was higher than accuracy of CE sequence. Our findings indicate a trend toward greater K1 for CE biopsy sites than for non-CE biopsy sites. It is known that a higher regional blood volume linked to a higher intratumoral microvessel density due to neoangiogesesis might be responsible for the higher AA PET tracers uptake in HGG compared to LGG (29, 30). Furthermore, the disruption of the blood–brain barrier is the mechanism suggested for the higher FET washout in HGG compared to LGG (31). However, the tumor accumulation of FDOPA in gliomas is also mediated through the specific LAT transport system, a tight bidirectional coupling of influx and efflux with obligatory exchange (32, 33). The estimates for the contribution of blood–brain barrier disruption and the specific LAT transport system contribution to the FDOPA uptake and washout in gliomas are not fully understood (34).

In our study, static parameters did not significantly differ between LGG and HGG. Regarding FDOPA uptake quantification using static parameters, conflicting results have been provided. While some authors found no association between FDOPA uptake and grading (8, 10, 11), others found higher FDOPA uptake values in HGG than in LGG (9, 12, 14, 16–18). Xiao et al. recently exhibited in a meta-analysis a pooled sensitivity of 0.71 and specificity of 0.86 for newly diagnosed gliomas grading using FDOPA PET static parameters (35). Our results confirmed that simplified quantification parameters have high specificity for gliomas grading. However, the current study showed that kinetic analysis provided a higher accuracy compared to static parameters for gliomas grading. These results are consistent with recent studies revealing that kinetic analysis markedly improved diagnostic performances for gliomas grading using FET PET imaging (21, 22), confirming that kinetic parameters provide different metabolic informations from that of static PET parameters. Regarding FET PET/CT, guidelines recently suggested that the standard method should be supplemented by the dynamic approach for non-invasive tumor grading of newly diagnosed gliomas with (23).

Our results showed that static and dynamic parameters were correlated with age. Carideo et al. already exhibited that age has an impact on FDOPA uptake (36).

The current study has several limitations. Only 14 patients were analyzed and only 9/32 samples were classified as LGG. The time range between FDOPA PET/CT and surgery was up to 110 days. However, only two patients had surgery more than 6 weeks after the FDOPA PET/CT and the pathological analysis revealed astrocytoma-IDH mutant for both patients. An IDIF was used for the PET kinetic modeling. However, recent studies also used an IDIF for quantifying FDOPA gliomas uptake (10, 15, 18, 37, 38). The information on dexamethasone or on medications with other steroids at the time of FDOPA PET/CT was not available (39). No partial volume correction was performed for the middle cerebral artery VOI. We compared FDOPA uptake parameters with IDH mutational status applying the IDH genotype of the whole specimen for each individual sample. Finally, the spatial accuracy of the neuronavigation-guided biopsies may have been impacted by brain shift after craniotomy, which could lead to brain shift. However, the biopsies were performed at the beginning of the surgical procedure before durotomy with Sedan needles, thus limiting the consequences of any such shift.

## Conclusions

The present study suggests an additive value of FDOPA PET/CT kinetic parameters for newly-diagnosed gliomas grading.

## Data Availability Statement

The original contributions presented in the study are included in the article/supplementary material, further inquiries can be directed to the corresponding author/s.

## Ethics Statement

The studies involving human participants were reviewed and approved by ethics committee of Ile de France 1. The patients/participants provided their written informed consent to participate in this study.

## Author Contributions

All authors made substantial contributions to the conception of the work, acquisition, interpretation of data, in drafting the work, and approved the submitted version.

## Conflict of Interest

The authors declare that the research was conducted in the absence of any commercial or financial relationships that could be construed as a potential conflict of interest.
